# Optimizing Evanescent Efficiency of Chalcogenide Tapered Fiber

**DOI:** 10.3390/ma15113834

**Published:** 2022-05-27

**Authors:** Xudong Zhao, Ni Yao, Xianghua Zhang, Lei Zhang, Guangming Tao, Zijian Li, Quan Liu, Xiujian Zhao, Yinsheng Xu

**Affiliations:** 1 State Key Laboratory of Silicate Materials for Architectures, Wuhan University of Technology, Wuhan 430070, China; 291035@whut.edu.cn (X.Z.); xzhang@univ-rennes1.fr (X.Z.); zijian_li@whut.edu.cn (Z.L.); quan_liu@whut.edu.cn (Q.L.); opluse@whut.edu.cn (X.Z.); 2 Research Center for Intelligent Sensing, Zhejiang Laboratory, Hangzhou 311121, China; zhang_lei@zju.edu.cn; 3 Laboratoire des Verres et Céramiques, UMR-CNRS 6226, Sciences Chimiques de Rennes, Université de Rennes 1, 35042 Rennes, France; 4 State Key Laboratory of Modern Optical Instrumentation, College of Optical Science and Engineering, Zhejiang University, Hangzhou 310027, China; 5 Wuhan National Laboratory for Optoelectronics, Huazhong University of Science and Technology, Wuhan 430074, China; tao@hust.edu.cn

**Keywords:** evanescent wave, tapered fiber, sensor, evanescent wave efficiency

## Abstract

Evanescent wave absorption-based mid-infrared chalcogenide fiber sensors have prominent advantages in multicomponent liquid and gas detection. In this work, a new approach of tapered-fiber geometry optimization was proposed, and the evanescent efficiency was also theoretically calculated to evaluate sensing performance. The influence of fiber geometry (waist radius (*R*_w_), taper length (*L*_t_), waist deformation) on the mode distribution, light transmittance (*T*), evanescent proportion (*T*_O_) and evanescent efficiency (*τ*) is discussed. Remarkably, the calculated results show that the evanescent efficiency can be over 10% via optimizing the waist radius and taper length. Generally, a better sensing performance based on tapered fiber can be achieved if the proportion of the *LP*_11_-like mode becomes higher or *R*_w_ becomes smaller. Furthermore, the radius of the waist boundary (*R*_L_) was introduced to analyze the waist deformation. Mode proportion is almost unchanged as the *R*_L_ increases, while *τ* is halved. In addition, the larger the micro taper is, the easier the taper process is. Herein, a longer waist can be obtained, resulting in larger sensing area which increases sensitivity greatly.

## 1. Introduction

Evanescent wave absorption-based fiber sensors have widespread applications ranging from biosensing to gas sensing. To date, various types of fiber sensors based on evanescent wave have been proposed, among which chalcogenide fiber possess unique advantages for its wide transmission window which covers the mid-infrared molecular fingerprint. In recent years, researchers from University of Rennes 1 [1,2,3], Ningbo University [4,5,6,7,8,9], Zhejiang University [10,11], and other institutions [12,13,14,15,16,17,18,19,20,21,22,23,24] have carried out extensive research on the mid-infrared evanescent wave absorption sensing technology, which proves that this technique can realize the real-time in situ qualitative and quantitative analysis of a variety of organic compounds. Dai et al. developed a gas sensor based on a four-hole suspended core As_2_S_3_ optical fiber, the sensitivity of which is less than 100 ppm for methane, and response time is estimated to be less than 20 s [8]. Romanova et al. prepared the Ge_26_As_17_Se_25_Te_32_ fiber loop for the detection of an anti-gel additive in diesel fuel. The absorption coefficients of the solution are well approximated by a linear function [13].

**Figure 1:**
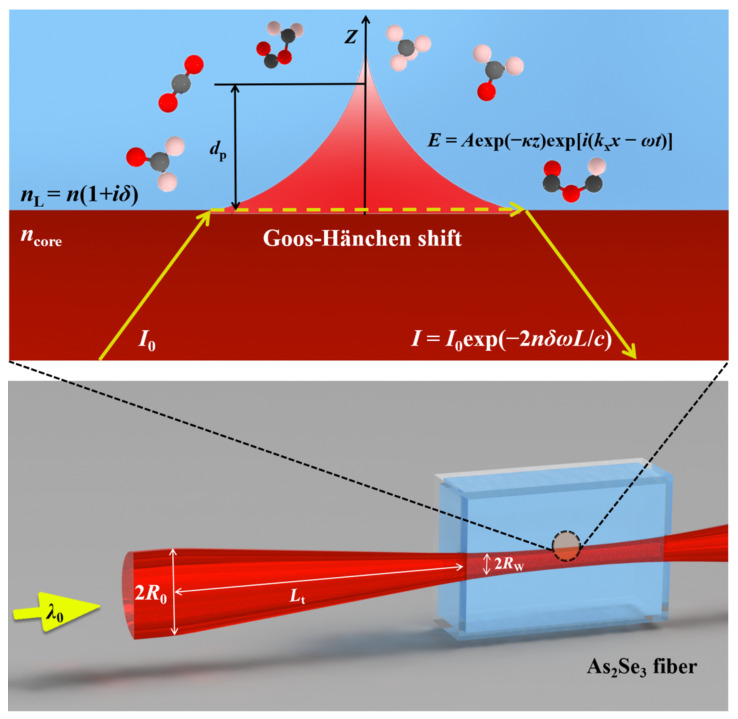
Mathematical model for numerical simulation.

Evanescent wave absorption sensing, based on the resonance absorption between the organic groups and the electromagnetic wave of a specific wavelength, can realize the molecule identification and concentration detection. As shown in Figure 1, the light is propagated through the fiber via successive total internal reflection, among which *E* = *A*exp(-*κz*)exp[*i*(*k*_x_*x-ωt*)] is the formula of the evanescent wave. The *A*exp(-*κ*z) and exp[*i*(*k*_x_*x-ωt*)] are the amplitude and phase of the evanescent wave, respectively. When the amplitude of the evanescent wave decays to 1/e of its maximum value, this position is defined as penetration depth, that is, *z* = 1/*κ*. The imaginary part of the refractive index is introduced to characterize the light absorption ability of external medium. It should be noted that the molecule identification and concentration detection are realized by analyzing the infrared spectra, including the intrinsic absorption and intensity of molecules.

How to improve the sensitivity of the fiber sensor has always been a research hotspot. The plasmonic-based fibers have high sensitivity in the visible region by using the surface plasmon resonance (SPR) effect of coating nanoparticles [22,23,24]. However, the SPR effect is not suitable for improving the sensitivity of infrared fibers. Geometry optimization is another way to improve the sensitivity of the fiber sensor. To date, varieties of the fiber geometry, such as tapered fiber [25], U-shaped fiber [26] and D-shaped fiber [27], have been widely studied, for these structures can strengthen the evanescent field greatly. Among them, the tapered fiber adjusts the mode distribution by reducing the core diameter to realize more evanescent wave energy existence in the boundary region. In the past years, waveguiding properties of tapered fiber have been extensively investigated theoretically, but mostly on adiabatic tapered silica fiber, which cannot transmit mid-infrared light [28,29,30]. Due to the great difference in optical properties between chalcogenide fiber and silica fiber, it is essential to carry out simulations for chalcogenide tapered fiber. To the best of our knowledge, no one has ever systematically studied the waveguiding properties of chalcogenide tapered fiber theoretically for sensing applications.

Here, we systematically investigate the evanescent field in different geometries of tapered fiber by using the finite-element-method-based full-vector solver COMSOL. Specifically, the electromagnetic wave frequency domain (ewfd) module is used for calculation. A general method for geometry design of chalcogenide tapered fiber is presented in this paper, which could be used for the tapered fiber optimization.

## 2. Basic Model

The mathematical model for our numerical simulation is illustrated in Figure 1, in which a tapered As_2_Se_3_ fiber with a radius of *R*_w_ is embedded in glucose solution. For simplicity, the radius (*R*_o_) of the untapered fiber is fixed at 100 μm, and the adjustable radius (*R*_w_) of the waist and the length (*L*_t_) of the cone are schematically shown in Figure 1.

To achieve high sensitivity for glucose, the input light wavelength for detection is assumed to be 7 μm, which corresponds to the position of the characteristic peak of glucose, of which the absorption coefficient is large, and the loss of this chalcogenide fiber at 7 μm is small. Another reason for this selection is that the cut-off radius corresponding to the same mode is different for different wavelengths. Among them, the shorter the wavelength, the smaller the corresponding mode cut-off radius and the smaller the radius of the optimized structure. The refractive indices of As_2_Se_3_ and water at 7 μm are 2.70 and 1.33, respectively.

**Figure 2:**
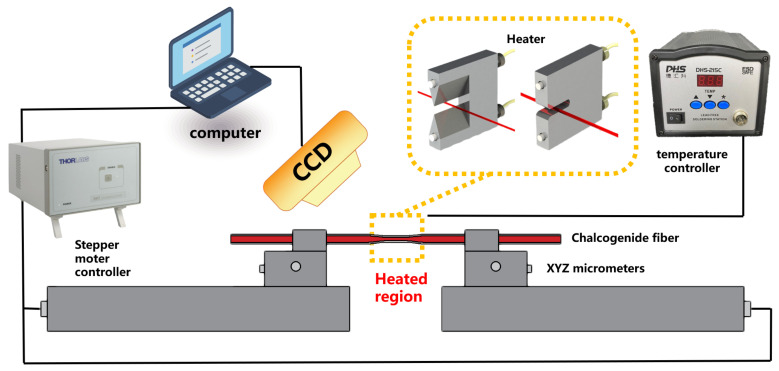
Experimental setup for the chalcogenide fiber tapering.

As shown in Figure 2, a potential fiber-puller system, which consists of an electrical heater, two motorized translation stages, a temperature controller, a CCD and a computer, can be used to draw the fiber taper. To achieve the flexible fiber geometry design, the modified resistance heating platform with different electrical heaters is controlled by a temperature controller. The chalcogenide fiber is fixed in fiber clamps and preheated before being pulled by the stepper motor controller. The diameter of the chalcogenide fiber is monitored in situ by CCD linked with a computer. Remarkably, the parameters chosen in this work have the potential to be realized by our homemade tapering platform.

The different geometries are investigated to evaluate the transmission losses, and the transmittance (*T*) can be described as follows:

(1) $$T={\sum \left(S_{21}\right)}^{2}$$

where *S*_21_ is the energy-output ratio of different modes. Typically, for the adiabatic taper, *T* is approximately equal to 1.

In order to ensure the accuracy of the calculation, it is necessary to ensure the accuracy of the energy at the incident end (*P*_in_) before calculating the evanescent efficiency. According to the wave optics, the energy at the incident end can be obtained by taking the surface integral of the Poynting vector. However, since this is a two-dimensional model, it is necessary to determine the height of the two-dimensional model first, as shown in the following formula:

(2) $$P_{\mathrm{in}}=\int_{l}S_{\mathrm{av}}dl\cdot H$$

where *l* is the incident end of the fiber, *S*_av_ is the Poynting vector and *H* refers to the height of the model. Significantly, the height is also applicable to the calculation of the following formula. In this model, *H* is calculated to be 1.939.

**Figure 3:**
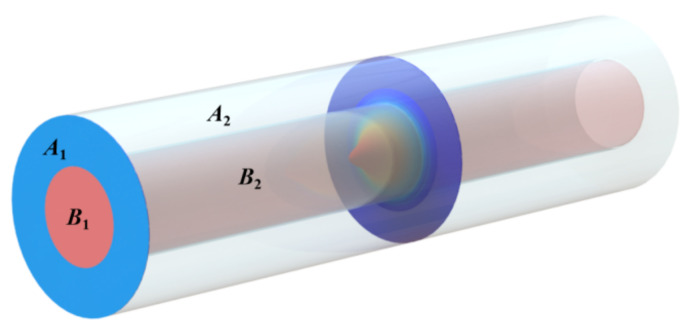
Calculation of the fractional power inside the fiber. *A*_1_ and *B*_1_ refer to the plane of core and cladding, respectively. *A*_2_ and *B*_2_ refer to the volume of liquid and core, respectively.

The profile of the power distribution is essential to evanescent-wave-based optical sensing. Taking the plane integral of the Poynting vector, Tong et al. calculated the fractional power inside the core (*T*_1_) under single mode condition and took it as the evaluation criteria of the fiber sensing performance as shown in Figure 3 [31]. Here, *A*_1_ and *B*_1_ refer to the planes of core and cladding, respectively. However, the evanescent field power changes periodically in the direction of light under the multimode condition due to mode interference. The formula calculating the evanescent field (*T*_o_) can be optimized by taking the volume integral of the average power. These two calculation methods are only applicable to adiabatic taper due to that the structural energy loss is negligible. To obtain arbitrary structural loss, the optimized evanescent proportion (*T*_o_) and evanescent efficiency (*τ*) are calculated as follows:

(3) $$T_{1}=\frac{\iint {}_{A_{1}}S_{\mathrm{av}}dB}{\iint {}_{A_{1}+B_{1}}S_{\mathrm{av}}dA}$$

(4) $$T_{O}=\frac{{∭}_{A_{2}}W_{\mathrm{av}}dB}{{∭}_{A_{2}+B_{2}}W_{\mathrm{av}}dA}$$

(5) $$\tau =P\cdot T_{O}=\left[{\sum \left(S_{21}\right)}^{2}\right]\cdot \frac{{∭}_{A_{2}}W_{\mathrm{av}}dB}{{∭}_{A_{2}+B_{2}}W_{\mathrm{av}}dA}$$

where *W*_av_ is the average energy density, and *A*_2_ and *B*_2_ refer to the volume of liquid and core marked in Figure 3. More formula details can be obtained from the Supplementary Materials.

## 3. Effect of Waist Radius

**Figure 4:**
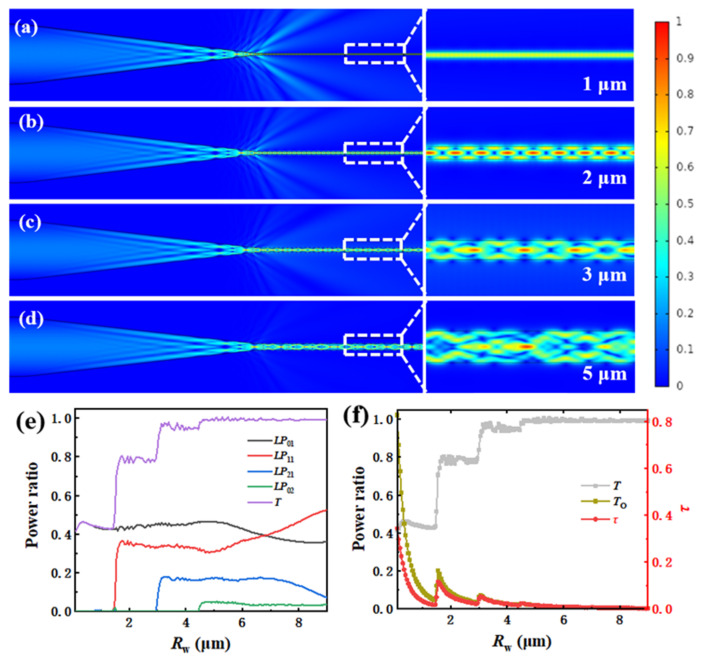
Schematic of the modal evolution in the transition region with (**a**) *R*_w_ = 1 μm; (**b**) *R*_w_ = 2 μm; (**c**) *R*_w_ = 3 μm; (**d**) *R*_w_ = 5 μm; (**e**) calculated modes proportion; (**f**) light transmittance, evanescent power ratio, evanescent efficiency in the waist with different *R*_w_.

The effect of *R*_w_ is first analyzed assuming a fixed *L*_t_ with 0.4 mm. The mode numbers of the fiber can accommodate are directly related to the normalized frequency:

(6) $$V=\kappa_{0}R_{w}\sqrt{n_{1}^{2}-n_{2}^{2}}$$

where *κ*_0_ is the vacuum wave number, *R*_w_ is the radius of the fiber and *n*_1_ and *n*_2_ are the core and cladding indices of the fiber, respectively. The electric field distributions for radius with 1 μm, 2 μm, 3 μm and 5 μm at 7 μm wavelength are shown in Figure 4. Generally, energy from fundamental mode transfer to high-order modes occurs during the transition from core to cladding because of the non-adiabaticity. The mode capacity decreases as the radius decreases further. A noticeable leakage is observed in the transition region between cone and waist when *R*_w_ with 1 μm can only accommodate the fundamental mode, as shown in Figure 4a. More details are shown in Figures S1 and S2. The calculation results of the light transmittance (*T*), *T*_o_, and the *τ* are 42.91%, 7.85% and 3.87%, respectively. Remarkably, the *τ* is much smaller than *T*_o_ due to the non-adiabatic taper. Figure 4b shows that the electric field distribution inside the waist changes periodically along the light propagation direction field when *R*_w_ is 2 μm. The position of the electromagnetic vibration peaks indicates that the *LP*_11_-like mode appears. Figure 4c demonstrates that the electric field distributions become more complex when *R*_w_ are 3 μm and 5 μm, respectively. It can be seen from Table 1 that *T* increases significantly from 42.91% to 98.72% and *T*_o_ decreases gradually from 7.85% to 1.67% with the increase in *R*_w_. Meanwhile, *τ* increases first and then decreases, indicating that some interesting phenomena occurred when *R*_w_ changed from 1 μm to 3 μm.

Figure 4e shows the power ratio of different modes of *R*_w_ from 0.8 μm to 9 μm. With the increasing of *R*_w_, the transmittance increases as a result of the increasing *V*, and *τ* is lower than *T*_o_ on the whole due to the optical loss caused by the non-adiabatic taper. When *R*_w_ is below 1.5 μm, only the fundamental mode can propagate in the waist. With *R*_w_ gradually decreasing, the electric field confinement of the waist gradually weakens, resulting in a larger evanescent field. Some peaks from the line of *T*_o_ and *τ* appeared when *R*_w_ was above 1.5 μm, corresponding to the cut-off radius of the higher-order mode. As *R*_w_ increases further, the proportion of modes in the waist remains the same, while the confinement of the waist on the electromagnetic field strengthens further, and *T*_o_ and *τ* start to decrease again. At the same time, as *T* approaches 1, the difference between *T*_o_ and *τ* gradually disappears (Figure 4f), which shows evanescent efficiency is suitable for both adiabatic and non-adiabatic taper.

The simulation shows that *T*_o_ and *τ* do not decrease monotonically with the *R*_w_ in the case of non-adiabatic taper. By increasing the taper length (*L*_t_), the non-adiabatic taper will gradually convert to an adiabatic taper. Next, the influence of *L*_t_ on light transmittance, evanescent field proportion and evanescent efficiency is analyzed.

## 4. Effect of the Length of Taper

**Figure 5:**
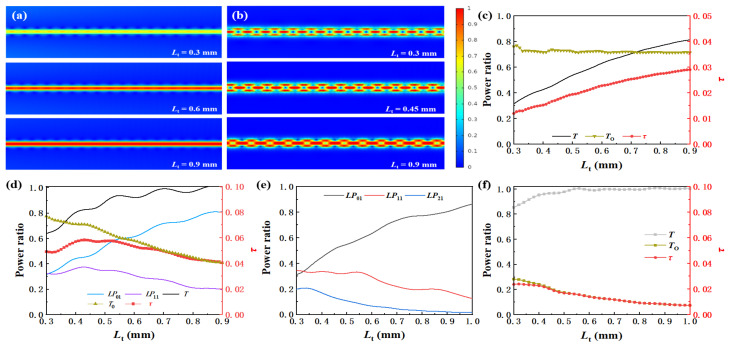
Calculated electric field in the waist as (**a**) *R*_w_ = 1.45 μm and (**b**) 2 μm; calculated modes proportion, light transmittance, evanescent power ratio, evanescent efficiency with different *L*_t_ as *R*_w_ fixed to (**c**) 1.45 μm and (**d**) 2 μm; calculated (**e**) modes proportion; (**f**) light transmittance, evanescent power ratio, evanescent efficiency in the waist as *R*_w_ is fixed at 4 μm.

Firstly, *R*_w_ is assumed to be fixed at 1.45 μm, on which condition the waist is single-mode fiber. Parametric scanning is applied to investigate the effect of *L*_t_ on waveguiding properties by increasing *L*_t_ from 0.3 mm to 0.9 mm with a 0.01 mm step. Figure 5a shows the electromagnetic field distribution in the waist with different *L*_t_, indicating that more energy is conserved when the cone is longer. Figure 5c demonstrates that more energy is coupled to the fundamental mode instead of the leaky mode with the increase in *L*_t_. The calculated *T*_o_ shows a downward trend due to the increasing fundamental mode. Meanwhile, *τ* shows an upward trend due to the significant increase in *T*.

Secondly, *R*_w_ is assumed to be fixed at 2 μm, on which condition the *LP*_11_-like mode can be transmitted in the waist. Figure 5b shows the electromagnetic field distribution in the waist. The energy of the *LP*_11_-like mode center at the boundary of the waist benefits the interaction between light and molecular groups. The calculated power proportion of different modes as a function of *L*_t_ is shown in Figure 5d. It is noticed that more energy couples to the fundamental mode as *L*_t_ increases, while the proportion of the *LP*_11_-like mode increases first and then decreases. The *T*, *T*_o_ and *τ* in the waist as a function of *L*_t_ are also presented in Figure 5d, respectively.

Finally, *R*_w_ is assumed to be fixed at 4 μm, on which condition three kinds of modes can transmit in the waist, as Figure 5e,f show. The *T*_o_ and *τ* are quite small because the light field is well restricted in the waist with a larger *R*_w_, indicating a poor sensing performance.

## 5. Optimization of Multimode Tapered Fiber

**Figure 6:**
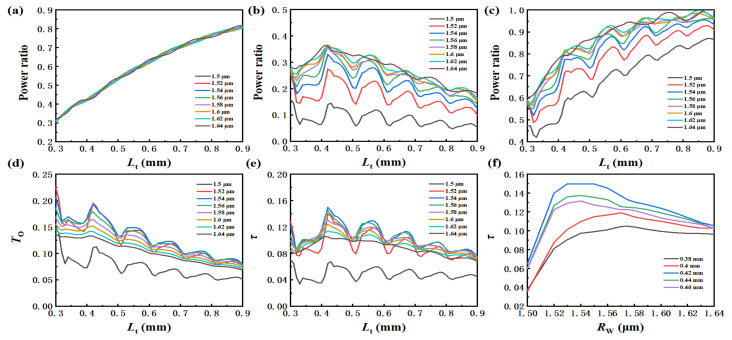
Calculated (**a**) fundamental mode proportion; (**b**) *LP*_11_-like mode proportion; (**c**) light transmittance; (**d**) evanescent proportion; (**e**) evanescent efficiency with different *L*_t_ as *R*_w_ changes from 1.5 to 1.61 μm; (**f**) calculated evanescent efficiency with different *R*_w_ as *L*_t_ changes from 0.38 to 0.46 mm.

In this section, different *R*_w_s which are close to the cut-off radius of the *LP*_11_-like mode are chosen for the *L*_t_ optimization calculation. Figure 6a–c give the calculated power proportion of *LP*_01_, *LP*_11_ and light transmittance, respectively. With the increase in *L*_t_, the proportion of *LP*_01_ increases, and the proportion of *LP*_11_ increases first and then decreases, which is consistent with the above results. Comparing Figure 6a,b,d,e, when *R*_w_ increases from 1.52 μm to 1.64 μm, the fundamental mode proportion stays unchangeable, and more *LP*_11_ transmits in the waist, while evanescent proportion decreases gradually. Notably, evanescent efficiency increases first and then decreases, which can be seen clearly in Figure 6f. Obviously, maximum evanescent efficiency exists at the place where the *LP*_11_ mode proportion does not reach its extreme. For the fiber with an original diameter of 100 μm in this simulation, when *R*_w_ and *L*_t_ are about 1.54 μm and 0.42 mm, respectively, the fiber sensor obtains the highest evanescent efficiency of 15%. Meanwhile, we can control the *R*_w_ in the range of 1.54~1.6 μm and the *L*_t_ in the range of 0.35~0.7 mm by the taper experiment, which is shown in Figure 2 to obtain a fiber sensor with evanescent efficiency higher than 10%.

By numerical simulation, we obtained the optimized geometry of tapered microfiber, which is very important for obtaining a high-precision and highly repeatable fiber sensor. It is noteworthy that this method can also be applied to the size design of U-shaped fiber, D-shaped fiber and other fibers with special shapes.

## 6. Analysis of Micro Deformation in Waist

The optimal size of the tapered As_2_Se_3_ fiber was achieved in Section 5, which could be realized by the fiber-puller system mentioned in Section 2. However, for the demand of liquid pool detection, in which condition a long waist for sensing is necessary, it is too hard to realize the geometry mentioned above. Thus, this geometry is only expected to be applied to single droplet detection, as the waist length is small. Therefore, we make a further calculation for the case that there is a micro cone in the waist.

**Figure 7:**
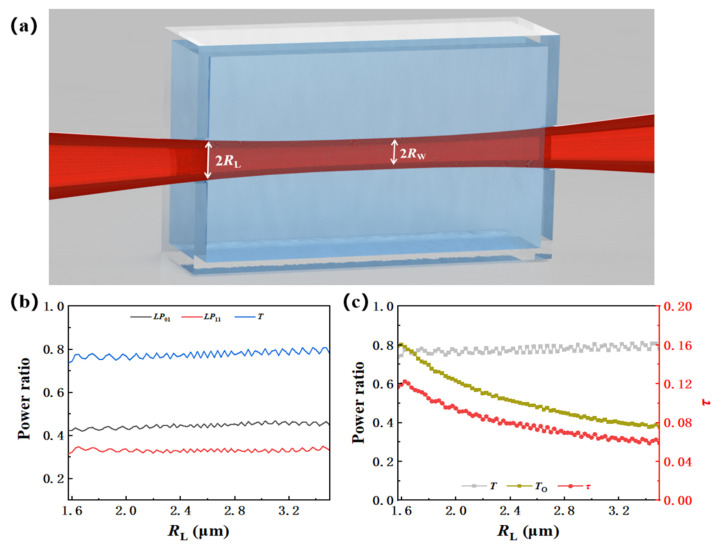
(**a**) Mathematical model; (**b**) calculated modes proportion; and (**c**) light transmittance, evanescent proportion and evanescent efficiency with different *R*_L_.

The waist region of the fiber with the micro taper is a part with changing radius, and the change is quite slow, so it can be approximated as a straight line with a small slope. Because the decreasing of fiber diameter will cause the change in mode, the light field in the micro cone is actually a process of coupling from high-order mode to low-order mode. Figure 7a gives the mathematical model for this section. The *R*_L_ increases from 1.56 μm to 3.5 μm to show the micro deformation of the fiber waist. Figure 7b,c give the mode proportion at *R*_w_ and the calculated *T*, *T*_o_ and *τ* in the waist, respectively. We find that with the increase in *R*_L_, the distribution of mode at *R*_w_ changes little, while the calculated *T*_o_ and *τ* decrease gradually. In other words, when the deformation is very small in the waist, the mode distribution is mainly determined by *R*_w_. This is because, on the one hand, *R*_w_ is fixed, while the mode field distribution in the optical fiber is mainly related to its radius. On the other hand, the taper in the waist area is very small, resulting in the mode conversion efficiency close to 1. Therefore, the mode proportion at *R*_w_ is almost unchanged at a different *R*_L_. Meanwhile, as the radius changes from *R*_L_ to *R*_w_, the equivalent radius of the waist area is greater than *R*_w_. The larger the radius is, the smaller the *τ* is (as shown in Figure 4). Although the evanescent efficiency decreases with *R*_L_, it can still be maintained above 6%.

In some cases, low evanescent efficiency does not mean poor sensing performance. As shown in Figure 1, raising the length of the sensing region can also increase the light absorbed by the molecular groups but needs more liquids. Although the deformation decreases the evanescent efficiency, it also reduces the requirement of precision in the fiber taper, which enables the manufacturing of fiber with a longer waist. Deformation is inevitable during the taper processing, but if we control it properly, the sensitivity can be improved.

As shown in Figure 2, different kinds of heaters will provide different temperature fields which could flexibly control the taper geometry. In addition, according to the literature [11], optimizing the taper drawing method can realize the long waist area. In practical terms, the fragility of the tapered fiber can be overcome by encapsulation with the UV curing adhesive in a liquid cell. However, we assume the loss the tapered fiber can be neglected in this simulation work. In practical tapered fiber, as the mode of light cannot be confined in the fiber when the radius is too small, the loss increased dramatically with the decrease in the waist radius, which limits the application of the tapered fiber with very small radius.

## 7. Conclusions

We systematically calculated the mode distribution in chalcogenide tapered fiber with flexible size design and defined the evanescent efficiency characterizing its sensing performance. Generally, *R*_w_ determines the mode capacity and the ability to limit light, and *L*_t_ determines the mode’s proportion and light transmittance. By optimizing *R*_w_ and *L*_t_, evanescent efficiency over 10% can be obtained for droplet liquid detection. For the liquid detection, although a high-precision taper cannot be achieved due to the inevitable micro deformation, we can lengthen the waist to obtain a better sensing performance. All in all, we give the general geometry design strategy of chalcogenide micro-nano fiber for sensing applications. While the tapered fiber has high sensitivity in liquid detection, there are still many challenges we have to face. The loss reduction and device encapsulation work can further promote the application of the chalcogenide fiber sensor. The experimental work on the taper fiber sensor is in progress.

## Figures and Tables

**Table 1:** Calculated light transmittance *T*, evanescent power ratio *T*_o_, evanescent efficiency *τ*.

*R*_w_ (μm)	Mode Evolution	*T* (%)	*T*_o_ (%)	*τ* (%)
1	single mode → multimode → single mode	42.91	7.85	3.87
2	single mode → multimode → multimode	76.62	7.09	5.43
3	single mode → multimode → multimode	83.78	5.01	4.20
5	single mode → multimode → multimode	98.72	1.67	1.65

## Data Availability

The raw data required to reproduce these results cannot be shared at this time as the data also form part of an ongoing study.

## Supplementary Materials

The following supporting information can be downloaded at: https://www.mdpi.com/article/10.3390/ma15113834/s1, Figure S1: Transmission of light in a non-adiabatic taper; Figure S2: The variation in electric field vibration from fundamental mode to higher-order mode and to fundamental mode in tapered fiber; 40–470 represent the different positions from incident end to exit end.
