# Supplementary material for: Optimizing Evanescent Efficiency of Chalcogenide Tapered Fiber
Source: Materials (Basel). 2022 May 27;15(11):3834. doi: 10.3390/ma15113834 (PMC9181228; doi:10.3390/ma15113834)
Supplement: Supplementary file 1 [file materials-15-03834-s001.zip › materials-1731915-supplementary.pdf]

materials

Formula Derivation

Light is a kind of electromagnetic wave with extremely short wavelength which follows Maxwell's equations. Since chalcogenide fiber is a uniform transparent medium without free charge and current, its differential form is as follows

$$\nabla \times \mathbf{E} = -\frac{\partial \mathbf{B}}{\partial t} \tag{S1}$$

Copyright: © 2022 by the authors. Licensee MDPI, Basel, Switzerland. This article is an open access article distributed under the terms and conditions of the Creative Commons Attribution (CC BY) license (https://creativecommons.org/licenses/by/4.0/).

$$\nabla \times \mathbf{H} = \frac{\partial \mathbf{D}}{\partial t} \tag{S2}$$

$$\nabla \cdot \mathbf{D} = 0 \tag{S3}$$

$$\nabla \cdot \mathbf{B} = 0 \tag{S4}$$

where **E** and **D** are the electric field strength and potential shift-vector respectively, **H** and **B** are the magnetic field strength and magnetic induction strength respectively, which meet the equations of $\mathbf{D} = \varepsilon\mathbf{E}$, $\mathbf{B} = \mu\mathbf{H}$, where ε and μ are the dielectric constant and permeability of chalcogenide glasses respectively. By taking the curl of (S1) and (S2) and using the vector expression, among which **A** is **E** or **H**, we can obtain the wave equation, as shown in (S5) and (S6). We can solve the wave equation with the help of different algorithms, such as FDTD, FEM, etc. Fortunately, various numerical calculation software based on these algorithms are developed to solve problems involved with computational optoelectronics.

$$\nabla^2 \mathbf{E} - \varepsilon\mu \frac{\partial^2 \mathbf{E}}{\partial t^2} + \nabla\left(\mathbf{E} \cdot \frac{\nabla \varepsilon}{\varepsilon}\right) = 0 \tag{S5}$$

$$\nabla^2 \mathbf{H} - \varepsilon\mu \frac{\partial^2 \mathbf{H}}{\partial t^2} + \left(\frac{\nabla \varepsilon}{\varepsilon}\right) \times \left(\nabla \times \mathbf{H}\right) = 0 \tag{S6}$$

By introducing the simple harmonic function $\mathbf{E} = \mathbf{E}_0 \exp[i(\mathbf{k}\cdot\mathbf{r} - \omega t)]$, the time and space components of the wave equation can be separated to obtain the waveguide field equation, as shown in (S7).

$$\nabla_t^2 \begin{bmatrix} \mathbf{E} \\ \mathbf{H} \end{bmatrix} + \chi^2 \begin{bmatrix} \mathbf{E} \\ \mathbf{H} \end{bmatrix} = 0 \tag{S7}$$

where ∇_t and χ are the transverse nabla operator and the transverse propagation constant, respectively. Since E_r, E_ϕ and H_r, H_ϕ can are expressed by E_z and H_z which are the components of **E** and **H**, Bessel equation can be obtained by bringing E_z and H_z into equation (S7), and E_z and H_z are obtained by select different Bessel functions according to the core and cladding, as shown in (S8).

$$\begin{bmatrix} E_z \\ H_z \end{bmatrix} = \begin{cases} \begin{bmatrix} A_1 \\ A_2 \end{bmatrix} J_l(UR_a)\exp\left[i(l\phi - \beta z)\right], (R_a \le 1) \\ \begin{bmatrix} B_1 \\ B_2 \end{bmatrix} K_l(WR_a)\exp\left[i(l\phi - \beta z)\right], (R_a > 1) \end{cases} \tag{S8}$$

where A_1, A_2, B_1 and B_2 are the constant to be solved by using electromagnetic wave boundary conditions. J_l and K_l are Bessel *J* function and *K* function of order *l* respectively. *U* and *W* are the transverse propagation constants of the electromagnetic field in the core and cladding, respectively. $R_a = r/a$ is the normalized radius and *a* is the radius of the core. By selecting the order of the Bessel function and combining the electromagnetic wave boundary continuity condition, we can obtain the eigenvalue equations of different modes.

For HE_{lm} and EH_{lm},

$$\left[\frac{J_l'(U)}{UJ_l(U)} + \frac{K_l'(W)}{WK_l(W)}\right] \bullet \left[\frac{J_l'(U)}{UJ_l(U)} + \frac{n_2^2 K_l'(W)}{n_1^2 WK_l(W)}\right] = \left(\frac{l\beta}{k_0 n_1}\right)^2 \left(\frac{1}{U^2} + \frac{1}{W^2}\right)^2 \tag{S9}$$

For TE_{0m},

$$\frac{J_0'(U)}{UJ_0(U)} + \frac{K_0'(W)}{WK_0(W)} = 0 \tag{S10}$$

For TM_{0m},

$$\frac{n_1^2 J_0'(U)}{n_2^2 UJ_0(U)} + \frac{K_0'(W)}{WK_0(W)} = 0 \tag{S11}$$

According to the order of Bessel function, we can obtain U_{lm} at the cut-off of different modes, and the linear relationship between longitudinal propagation constant of each mode β and normalized frequency *V* can be achieved. Moreover, Electromagnetic field equation of different modes can be achieved. By taking the surface integral of Poynting vector (S12), the distribution of energy at the section can be obtained, as shown in the following formula:

$$S = E \times H = (E_y H_z - E_z H_y)\mathbf{i} + (E_z H_x - E_x H_z)\mathbf{j} + (E_x H_y - E_y H_x)\mathbf{k} \tag{S12}$$

$$P_{\mathrm{core}} = a^2 \int_0^{2\pi} \int_0^1 S_z R_a \mathrm{d}R_a \mathrm{d}\varphi = \frac{n\pi a^2 A^2}{2} \sqrt{\frac{\varepsilon_0}{\mu_0}} \left[1 - \frac{J_{l-1}(W) J_{l+1}(W)}{J_l^2(W)}\right] \tag{S13}$$

$$P_{\mathrm{sur}} = a^2 \int_0^{2\pi} \int_1^{\infty} S_z R_a \mathrm{d}R_a \mathrm{d}\varphi = \frac{n\pi a^2 A^2}{2} \sqrt{\frac{\varepsilon_0}{\mu_0}} \left[\frac{K_{l-1}(U) K_{l+1}(U)}{K_l^2(U)} - 1\right] \tag{S14}$$

where P_{core} is the energy inside the core and P_{sur} is the energy outside the core. **This formula corresponds to Formula (S2) in the article.** In order to calculate the evanescent wave ratio, only the energy ratio outside the fiber core needs to be calculated. As shown in the following formula,

$$T_1 = \frac{P_{\text{sur}}}{P_{\text{core}} + P_{\text{sur}}} = \frac{\int_0^{2\pi} \int_1^{\infty} S_z R_a \mathrm{d}R_a \mathrm{d}\varphi}{\int_0^{2\pi} \int_0^{\infty} S_z R_a \mathrm{d}R_a \mathrm{d}\varphi} \tag{S15}$$

This formula corresponds to Formula (S3) in the article. Due to the influence of phase factor, the evanescent proportion of different sections is different. Therefore, axial parameters are introduced for calculation. As shown in the following formula:

$$T_o = \frac{P_{\text{sur}}^1}{P_{\text{core}}^1 + P_{\text{sur}}^1} = \frac{\sum_{l_1}^{l_2} \int_0^{2\pi} \int_1^{\infty} S_z R_a \mathrm{d}R_a \mathrm{d}\varphi}{\sum_{l_1}^{l_2} \int_0^{2\pi} \int_0^{\infty} S_z R_a \mathrm{d}R_a \mathrm{d}\varphi} \tag{S16}$$

This formula corresponds to Formula (S4) in the article. Then the formula of evanescent efficiency can be obtained by introducing light transmittance. COMSOL is a finite element calculation software. According to the finite element algorithm, it can directly solve the wave equation and obtain the electromagnetic field values of each point in the model. Therefore, the calculation accuracy can be guaranteed according to the evanescent efficiency formula based on traditional wave optics.

Moreover, the mode evolution in the fiber can be directly collected from COMSOL as shown in the Figures S1 and S2. Typically, great leakage mode can be observed in Figure S1.

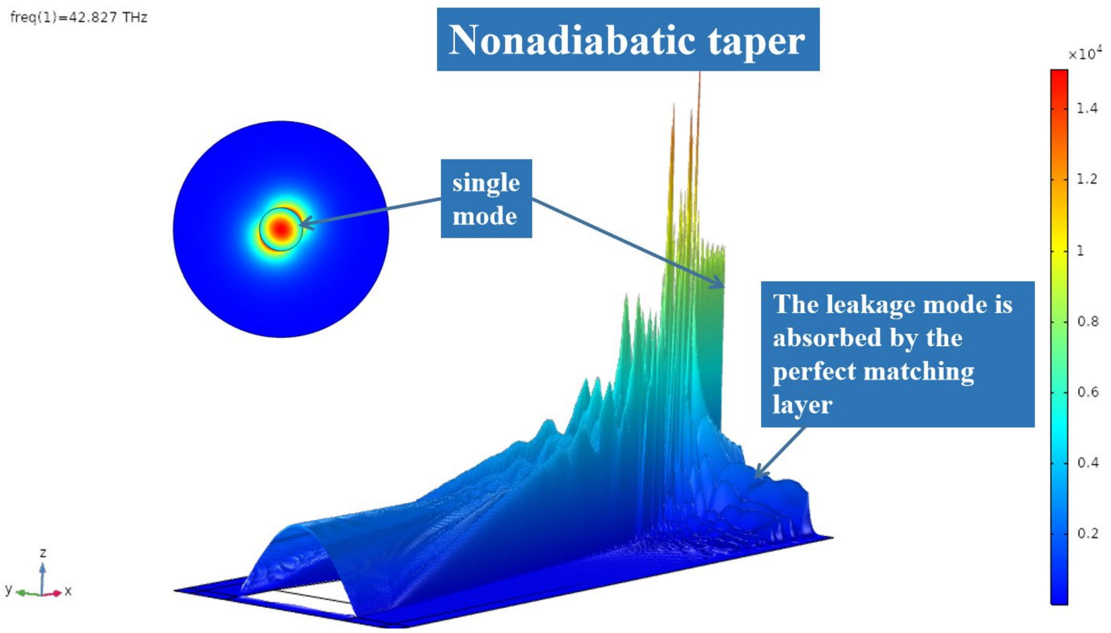

Figure S1. Transmission of light in a nonadiabatic taper.

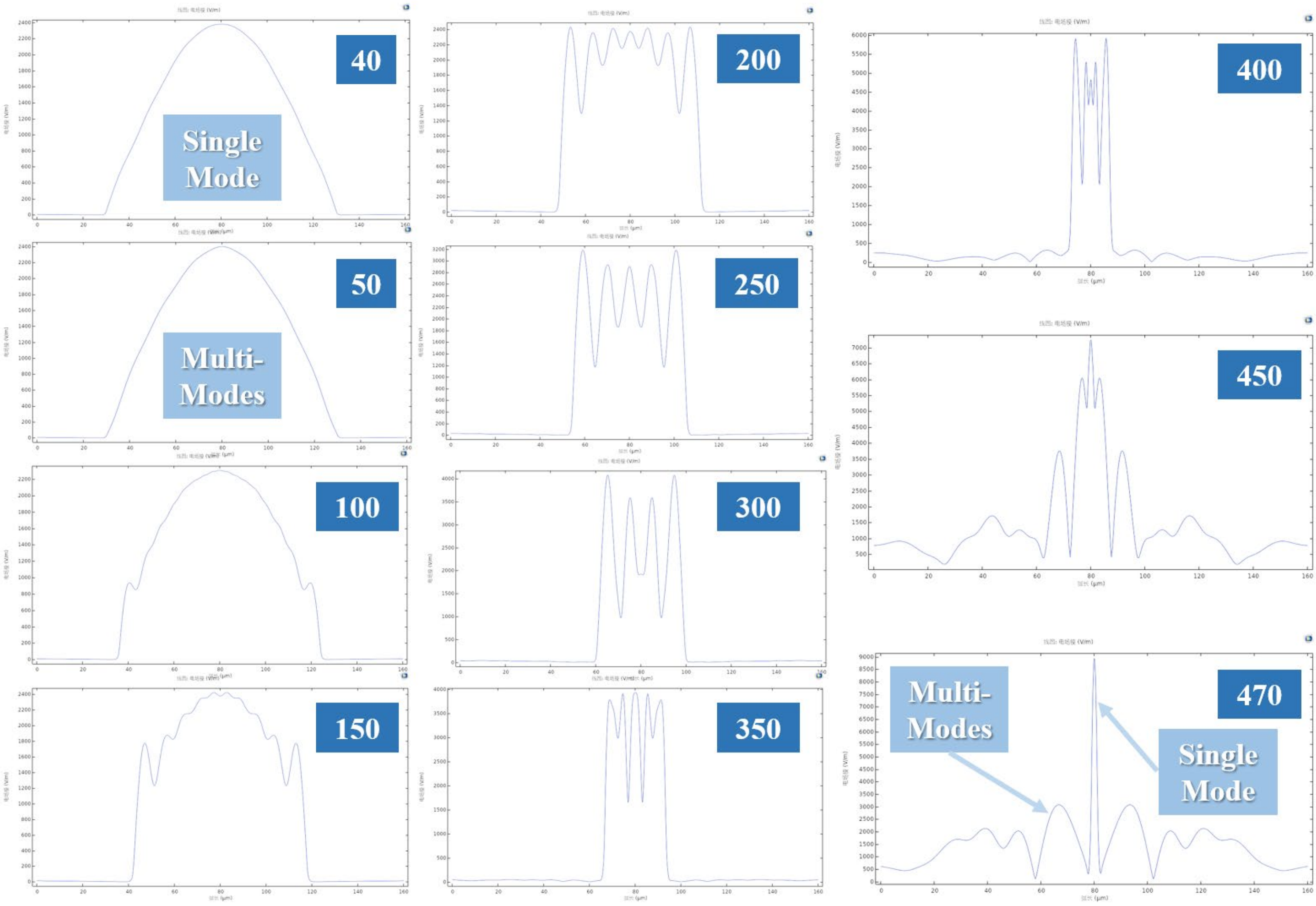

Figure S2. The variation of electric field vibration from fundamental mode to higher-order mode and to fundamental mode in tapered fiber, 40-470 represent the different positions from incident end to exit end.
